# Lifestyle as Risk Factor for Infectious Causes of Death in Young Dogs: A Retrospective Study in Southern Italy (2015–2017)

**DOI:** 10.1155/2020/6207297

**Published:** 2020-06-05

**Authors:** Lorena Cardillo, Giuseppe Piegari, Valentina Iovane, Maurizio Viscardi, Flora Alfano, Anna Cerrone, Ugo Pagnini, Serena Montagnaro, Giorgio Galiero, Giuseppe Pisanelli, Giovanna Fusco

**Affiliations:** ^1^Istituto Zooprofilattico Sperimentale del Mezzogiorno, Unit of Virology, Portici, Italy; ^2^Department of Veterinary Medicine and Animal Production, Unit of Pathology, University of Naples Federico II, Napoli, Italy; ^3^Department of Pharmacy (DIFARMA), University of Salerno, Fisciano, Italy; ^4^Istituto Zooprofilattico Sperimentale del Mezzogiorno, Unit of Diagnostics, Portici, Italy; ^5^Department of Veterinary Medicine and Animal Production, Unit of Microbiology, University of Naples Federico II, Napoli, Italy; ^6^Istituto Zooprofilattico Sperimentale del Mezzogiorno, Portici, Italy

## Abstract

Infectious diseases are a common cause of death in young dogs. Several factors are thought to predispose young dogs to microbiological infections. Identifying the cause of death is often a challenge, and broad diagnostic analysis is often needed. Here, we aimed to determine the infectious causes of death in young dogs aged up to 1 year, examining how it relates to age (under and over 6 months), lifestyle (owned versus ownerless), breed (purebred and crossbreed), and gender. A retrospective study was conducted in a 3-year period (2015–2017) on 138 dead dogs that had undergone necropsy and microbiological diagnostics. Enteritis and pneumonia were the most commonly observed lesions. Polymicrobism was more prevalent (62.3%) than single-agent infections and associated with a higher rate of generalised lesions. Ownerless dogs showed over a three-fold higher predisposition to viral coinfections than owned dogs. Above all, canine parvovirus was the most prevalent agent (77.5%), followed by canine coronavirus (31.1%) and canine adenovirus (23.9%); ownerless pups had a higher predisposition to these viruses. *Escherichia coli* (23.9%), *Clostridium perfringens* type A (18.1%), and *Enterococcus* spp. (8.7%) were the most commonly identified bacteria, which mostly involved in coinfections. A lower prevalence of CDV and *Clostridium perfringens* type A was observed in puppies under 6 months of age. In conclusion, this study is the first comprehensive survey on a wide panel of microbiological agents related to necropsy lesions. It lays the groundwork for future studies attempting to understand the circulation of infectious agents in a determined area.

## 1. Introduction

A correct and complete determination of the cause of death in young dogs is a challenge for veterinarian practitioners. A mere anatomopathological examination is often not sufficient to define a lesion's etiology. Puppies are susceptible to several viral and bacterial pathogens owing to the incomplete ability of their immature immune system [1]. In the first days of life, bacterial infections are described to be the prevalent cause of neonatal disease and death [2]; in contrast, at other ages, many factors have been attributed for outbreaks of viral diseases, including age, vaccination status, breed [3, 4], habitat [5], and seasons [6]. Stressful conditions due to overpopulation, high environmental contamination [7], lengthy travel for illegal importations, and lack of vaccination can create immune deficiency [8]. In this context, viral infections and bacterial superinfections can occur, and mixed infections are frequently detected [9, 10]. Polymicrobial infection recognises several etiopathogenetic mechanisms: (1) universally recognised virus-induced immunosuppression created by some agents such as canine parvovirus (CPV) leukopenia and disruption of the gastrointestinal barrier [1] or canine distemper virus (CDV) lymphopenia [11], which create a niche for the growth of other opportunistic pathogens; (2) the so-called primary with secondary infections, where the first agent creates the ideal condition needed for the colonisation and replication of the second one, e.g., “kennel cough,” where some respiratory viruses such as CDV, canine adenovirus type 2 (CAV-2), *Canid herpesvirus-1* (CHV-1), and others precede the secondary bacterial infection [12, 13]; (3) a condition characterised by concurrent infection of multiple agents to induce the disease [8].

In addition, an immune-compromised system can play a key role for systemic spread of localised infections in various ways, including extraintestinal diffusion of enteric pathogens, because of gut dysbiosis, either through the choledochus or via bacterial translocation from the lymph-hematic route [14–16], as described for coliform septicemia in dogs with viral-induced damage of intestinal epithelial cells during parvoviral infections [17]. In many cases, coinfectants can exacerbate clinical signs; thus, normally mild pathogens can cause severe diseases [18, 20]. Although vaccines for some pathogens that cause high mortality in pups have been produced, a failure of vaccination can occur, due to interference of high titres of maternal-derived antibodies (MDAs), incorrect vaccination protocols, high environmental contamination, or stressful conditions [21, 22]. The identification of specific causes of death has a fundamental epidemiologic role. Several studies have been conducted in the past, based only on anatomopathological lesions [23–26], and, more recently, a retrospective study was reported in the province of Rome (Italy), based on anatomohistopathological examinations and collateral exams [27]. To the authors' knowledge, there are no epidemiologic surveys on the causes of death for infectious diseases in young dogs performed in Southern Italy. Thus, the aim of this study was to identify infections and coinfections associated with macroscopic lesions in deceased dogs under 1 year of age, related to their age and lifestyle.

## 2. Materials and Methods

### 2.1. Subjects and Data Collection

Our study is a retrospective survey carried out at the Istituto Zooprofilattico del Mezzogiorno (IZSM) of Portici, Naples (Southern Italy), as part of our routine diagnostic activities aimed to verify the causes of death in owned and ownerless young dogs. From 511 dead dogs brought to the IZSM by veterinary practitioners, owners, or law enforcement officers in order to assess the cause of death, 138 fulfilled the criteria to be included in the study. These criteria included signs of death due to infectious diseases, confirmed by anatomopathological and chemical exams, complete panel for necropsy, and microbiological examinations; ages ranged from 0 to 12 months. Data included signalment, anamnesis, necropsy, and microbiologic reports, which were carried out over a 3-year period (January 2015 to December 2017) from the Informative System for Analysis Laboratory Management (S.I.G.L.A.) database of the IZSM.

For the content of this survey, the authorisation of the Ethical Committee was deemed unnecessary, according to national regulations.

### 2.2. Macroscopic Diagnosis of Cause of Death

All necropsies were performed in the necropsy room in the IZSM with a standard necropsy protocol codified by Piegari et al. [28, 29]. Approximatively, 2 cm^2^ of tissue samples and swabs were collected from deeper structures of the liver, lungs, brain, heart, and small intestine. Fecal swabs were collected from rectal ampulla. Specimens were processed for microbiological examinations as soon as possible.

### 2.3. Virological Analysis

#### 2.3.1. Nucleic Acid Extraction

Approximately, 2 mg of each tissue sample was collected and suspended in 2 mL of sterile phosphate buffer saline (PBS) in 2-mL tubes, homogenised with glass beads with the TissueLyser (Qiagen), and clarified by centrifugation for 5 min at 4000 rpm.

Fecal specimens were subjected to a previously described treatment, in which 100 mg of feces was suspended in 900 *µ*L of tissue lysis buffer (ATL), vortexed for 1-2 min, and incubated for 10 min at room temperature to obtain sedimentation of greater particles; finally, 600 *µ*L of supernatant was transferred to a 1.5-mL tube and incubated at 70°C for 10 min in a thermomixer (Eppendorf).

Aliquots of 200 *µ*L of both tissue and fecal supernatants were collected for nucleic acid extraction using the QIAsymphony automated system (Qiagen) and processed according to the manufacturer's protocol, eluted in 60 *µ*L, and stored at −80°C until use.

#### 2.3.2. PCR Protocols

Samples and fecal swabs were tested for the most common viruses that cause diarrhea, pneumonia, hepatitis, encephalitis, and myocarditis in young dogs, using PCR protocols reported in Table 1.

For molecular detection and characterisation of CPV variants (2a-2b-2c), real-time PCR was performed using different sets of primers and probes to amplify fragments encoding for capsid protein VP2, as described previously [30]. For CCoVs, the molecular detection and genotyping of CCoV-I and II were performed using a set of primers and probes that amplify fragments of ORF5 as described by Decaro et al. [32], and the 2 subtypes CCoV-IIa and IIb were characterised by the amplification of the gene encoding the spike (S) protein using the RT-PCR protocol reported by Decaro et al. [33]. Finally, for CAVs, the protocol used was based on a duplex real-time PCR assay for the simultaneous detection of types 1 and 2, amplifying a fragment of the hexon gene, as described by Dowgier et al. [40]. The characterisation of CDV samples was performed on the hemagglutinin (H) gene sequence, according to An et al. [41]. Next, 1 *µ*L of the amplification product was applied to the TapeStation 2200 (Agilent Technologies) using D1000 Screen Tape and Reagents (Agilent Technologies). The PCR products were purified using the MiniElute Reaction Cleanup kit (Qiagen) according to the manufacturer's protocol. Reactions were subjected to Sanger sequencing carried out by BigDye Terminator Cycle Sequencing Kit v.1.1 (Applied Biosystems). The amplification conditions used for the sequencing reactions included an initial denaturation at 95°C for 1 min, followed by 25 cycles of denaturation at 96°C for 10 s, annealing at 50°C for 5 s, and extension at 60°C for 4 min. Amplicons were then cleaned up using the DyeEx 2.0 Spin kit (Qiagen) following the manufacture's indications. The sequencing reactions were applied to a 3500 Genetic Analyzer capillary electrophoresis system (Applied Biosystems). The forward and reverse sequences were assembled using the Geneious R9 software package (Biomatter) and compared to analogous sequences in the BLAST genetic database (http://www.ncbi.nlm.nih.gov/Blast.cgi).

#### 2.3.3. Bacteriological Analysis

Bacteriological analyses were performed by validated standard methods. Briefly, swab samples from the first isolation of the liver, lungs, brain, and small intestine were plated on MacConkey agar, aerobically incubated at 37 ± 1°C for 24–48 h and 5% blood sheep agar, incubated at 37 ± 1°C for up to 72 h in an aerobic or anaerobic atmosphere. For *Campylobacter* spp., Skirrow agar was used, and plates were incubated at 42 ± 1°C for 24–48 h in microaerophilic atmosphere, containing 6% O_2_, 10% CO_2_, and 84% N_2_. Anaerobic atmosphere was created by adding a 2.5-L Oxoid AnaeroGen Sachets to an Oxoid AnaeroJar (2.5 L, Thermo Fisher Scientific). When appropriate, enrichment broths were used. *Salmonella* spp. were isolated using preenrichment Müller-Kauffmann tetreathionate-novobicin broth (MKTTn) incubated at 37 ± 1°C for 24 ± 3 h and Rappaport-Vassalidis soy broth (RVS) at 41.5 ± 1°C for 24 ± 3 h and subsequently plated on xylose lysine desoxycholate agar (XLD) and Brilliance Salmonella agar base (BSA), both incubated at 37 ± 1°C for 24–48 h. Single colonies were selected and plated on specific media according to the findings. For biochemical identification, the VITEK2 system was used (bioMérieux). Finally, PCR methods were used in order to characterise *C. perfringens* toxins [42]. According to the identified etiologic agent, 5 categories were created: no agent detected, pure viral infections, mixed viral infections, bacterial infections, and mixed viral-bacterial infections.

#### 2.3.4. Statistical Analysis

Univariate models were used. The variables considered were lifestyle, age, gender, and breed.

Data were analyzed with an online software for statistical computation (VassarStats, Vassar College). Prevalence was calculated at a 95% confidence level. A chi-squared test of association was used to obtain the statistical significance level between groups, and risk assessment was performed with the odds ratio (OR) test to confirm the difference between groups. Results were considered statistically significant with a *p* value <0.05 and an OR > 1.0.

## 3. Results

From January 2015 to December 2017, 511 necropsies and microbiological analyses were recorded. Data were obtained from the IZSM database, and a total of 138 dogs were identified to fulfil the inclusion criteria. These dogs were under 1 year of age and comprised of 53 females (38.4%), 78 males (56.5%), and 7 of unknown sex (5.1%). They belonged to 30 different breeds: 37 crossbreeds (26.8%) and 93 purebreds (67.3%). Of the purebreds, the following breeds were recorded: 12 Pomeranians (8.7%), 11 Maltese (7.9%), 8 Chow Chows (5.8%), 6 each English Bulldogs and Chihuahuas (4.3% for each), 5 each Labrador Retrievers and Yorkshire Terriers (3.6%), 4 Neapolitan Mastiffs (2.9%), 3 each for Fox Terriers, German Shepherds, Husky, Italian Mastiffs, Pointers and Poodles (2.1%), 2 each for Cavalier King Charles Spaniel, Pinscher and Shiba Inu (1.4%), 1 each for Maremma Shepherd, Pitbull, Jack Russell Terrier, Great Dane, English Setter, Beagle, Dogue de Bordeaux, Argentine Mastiff, Akita Inu, and Pug and Dachshund (0.7%). For 8 reports (5.8%), however, no information about the breed was available. Four variables were examined: “lifestyle,” which included 70 ownerless dogs (50.7%), 33 strays that were housed in shelters (47.1%), and 37 that were confiscated by law enforcement for illegal importation (52.8%), and 68 dogs who lived in a house with their owner (49.2%). The “age” variable included 109 dogs under 6 months (78.9%) and 29 dogs from 6–12 months old (21%).

### 3.1. Necropsy Examination

**Figure 1:**
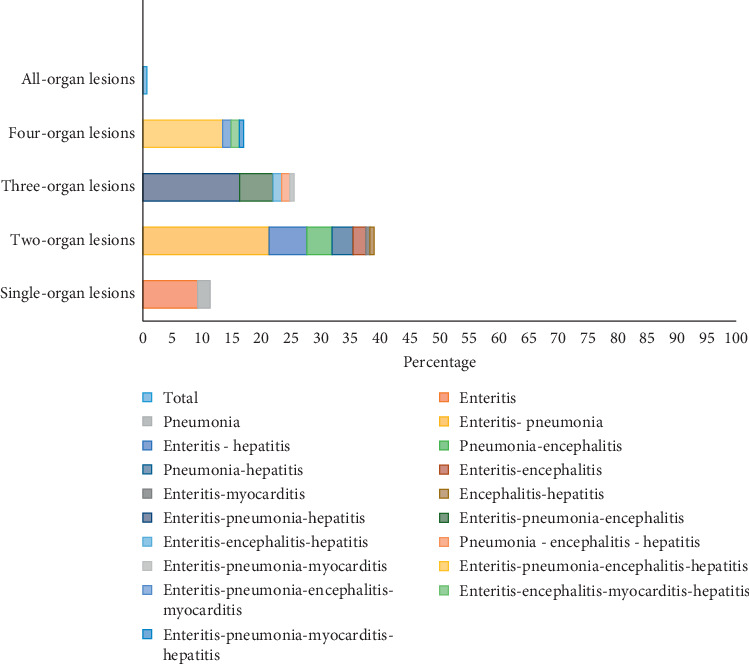
Necropsy reports. Prevalence of single-organ and multiorgan lesions.

Evaluation of the necropsy examination reports showed that the most frequently observed lesion was enteritis, which was found in 115 cases (83.3%), followed by pneumonia in 101 cases (73.1%), hepatitis in 65 cases (47.1%), encephalitis in 46 cases (33.3%), and finally myocarditis in 8 cases (5.8%). Multiorgan lesions were found to be more prevalent than those on a single organ; these lesion types were observed in 116 (84%) and 16 cases (11.6%), respectively (Figure 1). In 6 cases, no lesions were observed (4.3%). The organs were submitted to a broad microbiological analysis, and the chi-square analysis showed that there was a different trend in the distribution of the lesions in the organs related to microbiologic categories (*p* < 0.0001). Thus, while in single-organ lesions there was a higher prevalence of pure viral infections (43.7%), for cases with lesions in 2 or more organs, mixed viral-bacterial infections were more prevalent. The distribution of the lesions for the variables—lifestyle, age, gender, and breed—was investigated too (Table 2), and a different trend was observed between ownerless and owned dogs (*p*=0.013) as well as between crossbreed and purebred dogs (*p*=0.0026). Cases in which one or two organs were affected were more frequently observed in owned dogs, whereas instances with three-or-four-organ lesions were prevalent in the ownerless group of dogs. Regarding the breed variable, in crossbreeds, two organs were more commonly affected (56.7%); in contrast, purebreds had more diffuse lesions on three (30.1%) and four organs (21.5%). No difference was observed for age (*p*=0.1936) or gender variables (*p*=0.2328).

### 3.2. Microbiological Examination

Of the 138 young dogs examined, 132 (95.6%) had at least one pathogen. Overall, mixed infections, with two or more agents detected, represented 62.3% of cases (86/138), whereas the remaining 33.3% of dogs had only one agent. Pure viral and bacterial infections represented 71.7% (33/46) and 28.26% of single infections, respectively. Mixed viral, mixed bacterial, and mixed viral-bacterial infections represented 30.2% (26/86), 2.3% (2/86), and 42% of mixed infections, respectively.

A univariate analysis of the correlation of microbiological categories with the four variables considered in this study was conducted (Table 3). A significant difference was observed in ownerless dogs, which were approximatively 3-fold more predisposed to viral mixed infections (OR = 3.24; *p*=0.0117). In contrast, ownerless dogs were less frequently affected by bacterial infections than owned dogs (OR = 0.2; *p*=0.0120). For the breed variable, a statistically significant difference was observed in pure viral infections; thus, purebreds exhibited lower rates of pure viral infections than crossbreeds (OR = 0.22; *p*=0.0012). No difference was observed in the age or gender variables for any of the microbiological categories that were examined.

Our analysis of pathogens showed high prevalence of CPV (107/138; 77.5%), and it was frequently the causative agent of pure viral infections (27/33; 81.8%). Furthermore, it was also the most prevalent virus involved in viral coinfections (26/26; 100%) and viral-bacterial coinfections (55/58; 94.8%). High prevalence of canine coronaviruses was also observed (43/138; 31.1%). Although it was rarely observed in pure viral infections (4/33; 12.1%), canine coronaviruses were the second most prevalent agent involved in viral (18/26; 69.2%) and viral-bacterial (21/58; 36.2%) coinfections. A frequent association of this virus with CPV was observed in 79% (34/43) of CCoVs-positive samples. A modest circulation of CAVs and CDV was found in 23 (16.6%) and 19 (13.7%) pups, respectively. In contrast, CHV-1 and canine rotavirus (RV) had a very low prevalence: 5 (3.6%) and 2 cases (1.4%), respectively.

Bacterial infections were observed in the 52.9% of cases (73/138), mainly associated with viral pathogens (58/138; 42%). The following bacteria were found to be most prevalent: *Escherichia coli* was detected in 33 cases (23.9%), *Clostridium perfringens* type A (CPA) in 25 cases (18.1%), and *Enterococcus* spp. in 12 cases (8.7%). They were rarely identified as single pathogens but were often involved in mixed infections, mostly associated with CPV. Thus, *E. coli*, CPA, and *Enterococcus* spp. showed mixed infections with CPV in 75.7% (25/33), 80% (20/25), and in 100% of the cases, respectively. A very low prevalence was detected for other bacteria, which were mostly identified as opportunistic agents.

After examining bacterial and viral prevalence, infection risk was examined for the most frequently detected pathogens related to the four variables considered in the study (Table 4). Ownerless dogs showed a higher risk of infection to viral pathogens than owned dogs. The highest hazard risk was due to canine coronaviruses, with an odds ratio of 14.96 (*p* < 0.0001). When considering age as the variable, younger dogs, under 6 months, were less predisposed to CPV, CDV, and CPA than older dogs (with *p* values of 0.0444, 0.0007, and 0.0470, respectively). Finally, purebred dogs showed a higher prevalence for *E. coli* infections than crossbreeds (OR = 4.88; *p*=0.0137).

The relationship between pathogen and single/multiple organ (s) being affected was also investigated (Table 5). In single infections, CPV and CCoVs were associated with a higher degree of lesion generalisation: 51.8% (14/27) in 2 organs and 100% (4/4) of the cases in 3 organs, respectively, whereas *E. coli* was more associated with localised infections in 42.8% of the cases (3/7). Association of pathogens was observed to higher dissemination of the lesions.

Lastly, CPV, CCoV, and CAV variants were investigated. In 16 dogs (11.6%), a generalised CPV-vaccine strain was detected. In 15 of them, there was the copresence of other agents, and in 14 of them, there was also the CPV field strain. Within the 38.4% of positive samples (53/107), CPV-2a was found to be the prevalent antigenic variant, followed by CPV-2b, which was found in 28.2% of the cases (39/107). In contrast, no CPV-2c alone was detected. An association of 2 variants was observed in 15/107 cases (10.8%). More specifically, we found 8 cases of CPV-2b and 2c, 5 cases of CPV-2a and 2b, and 2 cases of CPV-2a and 2c. For canine coronaviruses, type II was the most prevalent, with 72.1% cases (31/43); of these, the CCoV-IIa variant was most common (28/31; 90.3%). Finally, CAV-2 had a higher prevalence than CAV-1, with 87.8% (29/33) and 12.1% (4/33) of the CAV infections, respectively; 1 case (3%) showed copresence of both strains.

Furthermore, in 16 CDV-positive samples, genetic characterisation was conducted on the hemagglutinin (H) glycoprotein, which showed a close match to Arctic-like lineages, both in owned and ownerless dogs.

## 4. Discussion

Breed, age, habitat, and stress are some of the risk factors that are known to predispose dogs to infections [3, 5, 8]. A compromised immune system, due to stress from overpopulation, long travels, or high levels of environmental contamination, can create conditions for viral infections and bacterial superinfections [9, 10]. Defining the causative agent of death on the basis of clinical and/or necropsy data without ancillary tests is quite difficult because of the similarity of symptoms and lesions between pathogens [43], and the presence of superinfectants can modify the original pathological findings of the diseases [44].

To assess infectious causes of death in dogs aged under 1 year of age, the circulation of agents in Southern Italy and whether age, lifestyle, gender, or breed can influence infections and relative lesion generalisation, a survey was conducted on 138 deceased pups.

A strong association between generalisation of lesions and microbiological infections was observed in this study (*p* < 0.0001). Necropsy examination showed that multiple-organ lesions were more frequently detected (84%) than single-organ lesions (11.6%), mostly due to viral-bacterial coinfections. Our results showed that multiple agents, with 2 or more pathogens detected, represented 62.31% of the cases. The presence of multiple agents creates conditions suitable for generalisation, because polymicrobism is reported to enhance other agent symptoms [18, 46] and diffusion of localised pathogens, as for coliform septicemia during parvoviral infection in puppies [17]. Enteritis was the most prevalent lesion (83.3%) due to the high detection of enteric agents. In 6 necropsies (4.3%), no macroscopic sign was observed. It is likely that sudden death occurred (unpublished data), and histologic alterations could have been present, given that in 5 of these 6 cases (83.3%) one or more pathogens were detected, but this aspect was not investigated in our study. Our study highlights the importance of lifestyle in the determination of infections. Young dogs are susceptible to several infectious diseases owing to the incomplete maturity of their immune system [1]; indeed, in dogs of 0-1 year of age, a higher rate of coinfection is observed than in any other age group [9]. Ownerless dogs showed a higher trend in lesion diffusion than dogs with owners (*p*=0.013). Environmental microbial contamination, lack of vaccination, overpopulation, and habitat are some of the risk factors reported to cause stress and predisposition to infections and coinfections [7, 8]. These conditions are frequently identified in shelters. Inappropriately constructed areas, dietary changes, and transport [47, 48], together with continuous introduction of new animals, make kennels a place where exposure, transmission, and susceptibility to infections are more evident [8, 49]. The same stressful conditions are identified in other contexts too, such as with the illegal trading of puppies or in stray dogs. Ownerless pups, in fact, showed a 3-fold higher risk for mixed viral infections (*p*=0.0117), but they had lower susceptibility to bacterial infections (OR = 0.2; *p*=0.0120). The breed was also identified as a significant risk factor for the generalisation of the lesions (*p*=0.0026). Indeed, purebreds showed a higher prevalence for multiple-organ lesions than crossbreeds, in which lesions were found with 56.7% of double-organ afflictions but a lower rate of pure viral infections (OR = 0.22; *p*=0.0012).

Our survey showed high prevalence of CPV (77.5%) and canine coronaviruses (31.1%) that are described to be the main causative agent of enteritis worldwide [50]. Eleni and colleagues in Central-South Italy demonstrated that CPV was the main causative agent of death in dogs under 1 year of age [27]. Other studies in diarrheic dogs showed lower prevalence of this virus, with a range from the 16% up to 54.3% [10, 51, 52]. CPV is characterised by high morbidity (100%) and mortality (up to 91%) [53]; thus, it is likely that the inclusion criteria of living dogs could be the main cause of difference with the other studies [10], making it challenging to compare the results. It was also observed that all the three parvoviral antigenic variants are circulating in Southern Italy; CPV-2a was the most prominent genotype, followed by CPV-2b and 2c [54].

CPV was also the most common virus involved in coinfections and detected in 100% of mixed viral infections and in 94.8% of viral-bacterial ones. The second most prevalent agent was canine coronavirus (31.15%), which is consistent with earlier studies [10, 50]. As described earlier, a strong association of CCoVs and CPV was observed [10] in 79% of the cases of coronavirus infection. When associated with other agents, coronavirus can cause either diarrhea by itself or exacerbate the symptoms of other viruses [18, 19, 55]. CAV was found in 23.9% of cases, and the CAV-2 strain was involved in 87.8% of the positive samples. Lower prevalence of CDV (13.7%), CHV-1 (3.6%), and rotavirus (1.4%) was observed. Molecular typing of CDVs was conducted on the H glycoprotein, which is considered the best target for the determination of the lineages due to its high variability [56]. In Italy, the Europe-1/South Africa-1 lineage is historically connected to CDV outbreaks; however, in the last 2 decades, the Arctic-like lineage is spreading throughout the country among both domestic and wild animals [57–60]. Our study corroborates these data, since the Arctic-like lineage was detected in CDV samples in both owned and ownerless dogs. This spread is speculated to be caused by dogs trading from East Europe, where the lineage was first detected [57, 59].

Lifestyle has been found to be an important risk factor for viral infections. Ownerless dogs showed a higher predisposition to CPV than dogs with owners (OR: 2.68; *p*=0.0331), CAV (OR: 4.16; *p*=0.0019), and CCoV, which represented the highest hazard for this variable (OR: 14.96; *p* < 0.0001), as assessed in other studies [8, 61, 62]. For the age variable, we did not observe the typical age trend of CPV infection, because these viruses are described to have the highest range of susceptibility from 3 to 6 months of age and are related to the decline of MDA [56, 63]. In our study, younger dogs were less involved in parvoviral infections (*p*=0.0444), as for CDV (*p*=0.0007) [4]. Another difference was observed for the breed and gender variable, which were, in general, associated with a higher risk for CPV and CCoV infections [4, 10]. We thus speculated about the role of lifestyle, which could be a more important risk factor than other factors, and the low immune system defense caused by several aforementioned factors can make older dogs even more easily affected than younger dogs [63–65].

Bacteria, mainly represented by enteropathogens, were rarely detected in pure infections (10.8%), and they were mostly associated with viruses in mixed infections (42%). Above all, *E. coli* (23.9%), CPA (18.1%), and *Enterococcus* spp. (8.7%) were the most common bacteria, followed by low percentages of other opportunistic bacteria. Since many of them belong to normal enteric microflora, they were considered only when toxigenic or in cases of localisation other than the gut. Dismicrobism, immunosuppression, and drug resistance are the predisposing causes for the passage of the bacteria from the gut to bile ducts and via the lymph-hematic route [14–16] that can lead to bacteremia [66], as for overpopulation, high environmental contamination, and viral copresence. This causes young dogs to have more exposure to environmental bacteria; moreover, especially when vaccination is not provided, they are predisposed to infection and superinfection, given a lack of a completely competent immune system [9].

## 5. Conclusions

To the authors' knowledge, this is the first comprehensive survey on the circulation of several infectious agents in Southern Italy related to necropsy examination. Our results indicate that lifestyle is an important risk factor for several viral pathogens and their generalisation, mostly identified in ownerless dogs that live in overcrowded habitat or are in contact with a stressful environment and intense conditions due to long travels. High circulation of enteric pathogens has been detected, with relative enteritis lesions in necropsy examination, mostly identified in CPV and CCoV and frequently involved in coinfections. CPV has been found as the most common pathogen involved in viral and viral-bacterial coinfections, highlighting its role in the suppression of the immune system.

In conclusion, the application of a broad microbiologic diagnostic panels for the identification of infectious agents that are responsible for the death of young dogs is an important tool for understanding the presence of infectious agents in a determined area and to clarify their epidemiological role in the genesis of diseases that lead to death. In addition, it can also be used as support for *intra vitam* diagnosis and in the therapeutic approach of veterinarian practitioners.

## Figures and Tables

**Table 1:** PCR protocols used for the detection and characterisation of viral pathogens.

Agent	Test and references
Canine parvovirus (CPV)	Real-time PCR, screening CPV/FPLV [30]
	Real-time PCR, CPV-2 based vaccine/CPV field strain [31]
	Real-time PCR, antigenic variants CPV-2a/2b and CPV-2b/2c [32]
Canine coronaviruses (CCoVs)	Real-time RT-PCR, screening CCoVs [33]
	Real-time RT-PCR, genotypes CCoV-I/CCoV-II [34]
	Real-time RT-PCR, subtypes CCoV-IIa/CCoV-IIb [35]
Canid herpesvirus 1 (CHV-1)	Real-time PCR [36]
Canine distemper virus (CDV)	Real-time RT-PCR [37]
	RT-PCR [41]
Rotavirus (RV)	Real-time RT-PCR [38]
Canine adenoviruses (CAV-1 and CAV-2)	PCR [39]
	Real-time PCR [40]

**Table 2:** Results of univariate analyses showing the distribution of the lesions in the organs related to the studied variables.

Variables		No lesion		1 organ		2 organs		3 organs		4 organs		5 organs		*χ* ^2^	*p*
		*n*	(%)	*n*	(%)	*n*	(%)	*n*	(%)	*n*	(%)	*n*	(%)		
Lifestyle	Ownerless	0	—	6	8.57	24	34.28	24	34.28	16	22.85	0	—	10.348	0.013
	Onwed	6	100	10	14.7	31	45.58	12	17.64	8	11.76	1	1.47		

Age	<6 months	5	4.58	12	11	41	37.61	33	30.27	17	15.59	1	0.91	1.69	0.1936
	>6 months	1	3.44	4	13.79	14	48.27	3	10.34	7	24.13	0	—		

Gender	Male	1	1.2	10	12.82	31	39.74	22	28.2	14	17.94	0	—	5.58	0.2328
	Female	4	7.5	6	11.32	18	33.96	14	26.41	10	18.86	1	1.88		

Breed	Purebred	4	4.3	12	12.9	28	30.1	28	30.1	20	21.5	1	1.07	16.36	0.0026
	Crossbreed	1	2.7	4	10.8	21	56.75	8	21.62	3	8.1	0	—		

**Table 3:** Correlation of the categories of infection to lifestyle, age, gender, and breed.

Category of infection	Variable	*χ* ^2^	*p*	OR	95% CI
Pure viral	Lifestyle	1.67	0.1962	0.54	0.25–1.21
	Age	0.04	0.8313	1.26	0.46–3.42
	Gender	0.19	0.5094	0.74	0.31–1.77
	Bacterial	Lifestyle	1.67	0.1962	0.54	0.25–1.21
Age		0.04	0.8313	1.26	0.46–3.42
Gender		0.19	0.5094	0.74	0.31–1.77
Breed		**9.91**	**0.0012**	**0.22**	**0.08–0.5**

Mixed viral	Lifestyle	6.35	0.0117	3.24	1.26–8.33
	Age	0.31	0.5776	0.66	0.33–1.54
	Gender	0.11	0.5873	0.79	0.34–1.82
	Breed	0.12	0.5591	1.32	0.51–3.43

Bacterial	Lifestyle	**6.31**	**0.0120**	**0.2**	**0.05–0.77**
	Age	2.07	0.1497	4.12	0.51–32.76
	Gender	0.77	0.2546	2.01	0.60–6.69
	Breed	2.84	0.0788	6.37	0.8–50.39

Mixed viral-bacterial	Lifestyle	3.67	0.0551	1.95	0.98–3.88
	Age	1.4	0.2355	0.6	0.26–1.38
	Gender	0.01	0.9276	1.03	0.5–2.09
	Breed	1.54	0.1531	1.54	0.8–3.98

**Table 4:** Results of the univariate analyses showing the association of the variables to the risk of infection for the most frequently detected pathogens.

Variables	Pathogen	*χ* ^2^	*p* value	OR	95% CI
Lifestyle	CPV	**4.54**	**0.0331**	**2.68**	**1.15–6.23**
	CCoVs	**33.27**	**<0.0001**	**14.96**	**5.36–41.69**
	CAVs	**9.6**	**0.0019**	**4.16**	**1.71–10.09**
	CDV	2	0.1572	2.35	0.83–6.61
	*E. coli*	1.67	0.1383	0.54	0.24–1.21
	CPA	0.13	0.5605	1.29	0.54–3.09
	*Enterococcus* spp.	0.06	0.5825	1.4	0.42–4.64

Age	CPV	**4.04**	**0.0444**	**0.2**	**0.04–0.91**
	CCoVs	2.55	0.1102	2.56	0.9–7.27
	CAVs	2.83	0.0925	3.29	0.92–11.68
	CDV	**11.15**	**0.0007**	**0.17**	**0.06–0.47**
	*E.coli*	0.05	0.6475	1.26	0.46–3.42
	CPA	**3.21**	**0.0470**	**0.38**	**0.14–0.98**
	*Enterococcus* spp.	—	—	—	—

Gender	CPV	0.19	0.5190	0.75	0.32–1.75
	CCoVs	0.52	0.3645	1.41	0.66–3.02
	CAVs	0.05	0.6627	0.83	0.37–1.87
	CDV	0.84	0.2464	0.56	0.21–1.49
	*E.coli*	0.19	0.5190	1.31	0.57–3.03
	CPA	2.23	0.0884	0.45	0.18–1.12
	*Enterococcus* spp.	0.05	0.9287	0.94	0.28–3.15

Breed	CPV	0.02	0.7076	0.83	0.33–2.09
	CCoVs	2.39	0.0841	2.18	0.9–5.31
	CAVs	2.65	0.0706	2.61	0.92–7.43
	CDV	0.25	0.4416	1.58	0.48–5.14
	*E.coli*	**5.89**	**0.0137**	**4.88**	**1.38–17.22**
	CPA	0.08	0.7810	1.15	0.41–3.2
	*Enterococcus* spp.	1.65	0.1387	4.8	0.6–38.82

**Table 5:** Correlation between dissemination of the lesions and the prevalent pathogens.

Pathogen	Total	No organ (6)		1 organ (16)		2 organs (55)		3 organs (36)		4 organs (24)		5 organs (1)	
		*n*	(%)	*n*	(%)	*n*	(%)	*n*	(%)	*n*	(%)	*n*	(%)
CPV	27	2	7.4	7	25.9	14	51.85	3	11.11	—	—	1	3.7
*E. coli*	7	—	—	3	42.85	1	14.28	2	28.57	1	14.28	—	—
CCoVs	4	—	—	—	—	—	—	4	100	—	—	—	—
CPV-CCoVs	13	—	—	3	23.07	8	61.53	1	7.69	2	15.38	—	—
CPV-*E. coli*	13	3	23.07	1	7.69	3	23.07	4	30.76	2	15.38	—	—
CPV-CAVs	10	—	—	—	—	7	70	3	30	—	—	—	—
CPV-CDV	9	—	—	—	—	2	22.22	1	11.11	6	66.66	—	—
CPV-CCoVs-CAVs	22	—	—	—	—	9	40.91	9	40.91	4	18.18	—	—
CPV-CCoVs-CHV	3	—	—	—	—	—		—	—	3	100	—	—
No agent	5	1	20	—	—	2	40	2	40	—	—	—	—
Others	24	—	—	2	8.33	9	37.5	7	29.16	6	25	—	—

## Data Availability

The data used to support the findings of this study are included within the supplementary information file.

## Supplementary Materials

Supplementary Materials The supplementary material contains raw data on age, breed, gender, and lifestyle of the dogs and the results of microbiological and necropsy examination.
